# The Therapeutic Effect of Electroacupuncture Therapy for Ischemic Stroke

**DOI:** 10.1155/2020/6415083

**Published:** 2020-09-29

**Authors:** Bao-Hai Yu, Ying Xing, Feng Zhang

**Affiliations:** ^1^Department of Radiology, The Third Hospital of Hebei Medical University, Shijiazhuang 050051, China; ^2^Department of Rehabilitation Medicine, The Third Hospital of Hebei Medical University, Shijiazhuang 050051, China; ^3^Hebei Provincial Orthopedic Biomechanics Key Laboratory, The Third Hospital of Hebei Medical University, Shijiazhuang 050051, China

## Abstract

Electroacupuncture (EA) stimulation is a supplementary therapy and commonly applied in treatment of ischemic stroke in clinic. Stroke is an important cause of long-term disability in individuals in both developing and developed countries. In our review, we show the application of EA stimulation for apoplectic pain, limbs spasticity, blood flow interruption, depression, swallowing dysfunction, aphasia, urinary incontinence, cognition and memory impairment, and constipation following stroke in patients and the related mechanisms in animals. The effectiveness of EA involves with acupoints, intensity, intervals, and duration of intervention for treatment of stroke. The combination of EA and common rehabilitation treatment may exert better effect compared with EA alone. In summary, EA might provide a potential treatment strategy for treating apoplectic patients in clinic.

## 1. Introduction

Ischemic stroke is a usual cerebrovascular illness and a leading cause of disabilities and death worldwide, accounting for approximately 87% of all stroke patients. Also known as brain attack, patients may suddenly suffer from incoherent speech, paralysis, or loss of vision owing to interrupting blood flow (ischemia) resulted from embolism or thrombosis [1]. Ischemic stroke induces a decrease in cerebral blood flow, which is enough to impair normal cellular function [2, 3]. Fast reperfusion is a crucial therapy method for patients with acute ischemic stroke but usually results in cerebral ischemia/reperfusion injury [4]. Therefore, a feasible therapeutic method that attenuates the poststroke neural deficits still is essential in the clinical setting. Acupuncture has been used in treating cerebral diseases and mental disorders for a long time [5, 6]. Electroacupuncture (EA) is another type of acupuncture, originating from the combination of acupuncture and electrical stimulation. As a relatively feasible, simple, and cheap therapy, it is commonly accepted by stroke patients in clinic [7]. The clinical effectiveness of EA in stroke rehabilitation has been demonstrated in numerous studies [8–10].

## 2. The Therapeutic Effect of EA for Ischemic Stroke in Experimental Animals

### 2.1. The Effect of EA on Neurocytes in Animals

EA may prompt neuronal regeneration, migration of newborn neuron, and their maturation in the ischemic brain striatum of rats [11]. EA stimulation at Dazhui (GV 14) and Baihui (GV 20) four-day postischemia (subacute stage) can enhance astrogliosis and neurogenesis, which likely contributed to long-term functional recovery after focal cerebral ischemia [12]. Deng et al. show that EA stimulation may increase new projections and axon regeneration from the corticospinal tract at 28 d following ischemic stroke in rats [13]. The vagus dorsal motor nucleus, as the largest origin of parasympathetic preganglionic neurons, may be activated by EA in the lower brainstem, and parasympathetic dysfunction may inhibit these abovementioned alterations, indicating that EA may be an alternative therapy to activate the parasympathetic nervous system after stroke [14]. Han et al. indicate that EA may be involved in activation of astrocytes in peri-ischemic brain, promotion of the recovery of behavioral deficits, and prevention of excess reactive gliosis after ischemic stroke [15]. EA stimulation at Renzhong may exert benefits in improvement of motor function and the motor cortical excitability following ischemic stroke [16]. Si et al. show that EA may prompt somatosensory evoked potential of rats following ischemic stroke [17]. EA treatment at points of Quchi and Zusanli can increase the functional connectivity between the ipsilateral motor cortex and the motor function-related brain regions, consisting of the motor cortex, striatum, and sensory cortex in focal ischemic rats [18].

### 2.2. The Effect of EA on Cerebral Angiogenesis and Blood Flow in Animals

Du et al. suggest that EA might play a crucial role on promotion of angiogenesis in cerebral ischemic rats [19]. Shi et al. also show that EA at Shuigou (GV26) enhances angiogenesis and establishment of collateral circulation and prompts neurological function [20]. Increased expression of apelin-APJ protein and mRNA induced by EA (15 Hz, 2 mA) applied at Shuigou (GV 26) exerted a crucial role in cerebral ischemic rats which maybe involved in facilitated collateral circulation and blood vessel regeneration [21]. EA at Yin meridian acupoints can significantly facilitate neurobehavioural functional recovery, which is associated with increased vascular density and enhanced vascular endothelial growth factor (VEGF) expression and protein kinase B/endothelial nitric oxide synthase (Akt/eNOS) phosphorylation in the peri-ischemia cortex of rats [22]. Liu et al. show that EA can balance miRNA levels, such as mir-328 and mir-126, so as to promote angiogenesis in ischemic cortex via regulating expression of VEGF family genes and proteins [23]. Furthermore, Hsieh et al. show that EA with a frequency of 2 and 15 Hz at Zusanli acupoints in both two legs may lead to the enhancement of cerebral blood flow in normal or ischemic stroke rat [24]. Zhou et al. demonstrate that EA intervention may exert brain protection via rapidly upregulating blood flow of the infarction region [25].

### 2.3. The Effect of EA on Improving Motor Dysfunction of Animals

EA with low frequency at Shuigou acupoint may exert obvious effect to prompt motor functional recovery in rats following ischemic stroke [26]. Liu and Lai demonstrate that EA plays a critical role in treatment of ischemic brain injury in the early stage of stroke and may effectively alleviate ischemic pathological damage, infarct volume, and neurologic deficit [27]. Liu et al. also suggest that EA at the points of ST36 and LI11 may reduce the infarct volumes, alleviate neurological deficit, and improve motor dysfunction [28]. It should be noted that many clinical research has identified that there can be no direct evidence/relationship between the infarct volume change/difference and the functional recovery. Therefore, rationale of reduction in the infarct volume by electroacupuncture therapy is not clear.

### 2.4. The Effect of EA on Autophagy and Apoptosis in Animals

EA treatment at points of Quchi and Zusanli can exert protective effects in rats with cerebral ischemia/reperfusion injury, associating with the inhibition of neuronal autophagy and apoptosis through activating the PI3K/AKT/mTOR pathway [29]. EA may also effectively alleviate central poststroke pain and suppress autophagy in the hippocampus through reducing *β*-catenin/COX-2 protein levels [30]. Xing et al. show that the neuroprotective effect induced by EA treatment against cell apoptosis in ischemic brain might associated with upregulation of midkine and regulation of ERK/JNK/*p*38 signal pathway [31].

### 2.5. The Effect of EA on Cerebral Edema and Blood-Brain Barrier (BBB) in Animals

Jung et al. show that EA pretreatment alleviates cerebral edema and blood-brain barrier (BBB) destruction, which may improve neural function. The BBB recovery by EA pretreatment might be associated with reduction of NOX4 expression and ROS generation [32]. Zhang et al. show that EA may improve brain edema in rats with ischemic stroke [33]. The inhibition of cerebral edema and BBB permeability induced by EA pretreatment was correlated with inhibition of *p*-caveolin-1 expression and alleviation of tight junction protein degradation and in the endothelial cells [34].

### 2.6. The Effect of EA on Other Aspects in Animals

Acupuncture treatment is a crucial part of Chinese traditional medicine and its feasible analgesic effect is widely accepted worldwide [35]. Lin et al. show that EA at Shenting and Baihui acupoints exerts a beneficial effect in promoting the cognitive function recovery after cerebral ischemic stroke [36]. EA may decrease the episodes of spreading depression after cerebral ischemic stroke, which may involve in the reduction of infarct volume of ischemic brain [36]. EA at Shuigou (GV26) significantly improved the neurological deficit symptoms in rats with ischemic stroke, which may be involved in upregulating Wnt7a and LEF1 proteins and mRNAs levels and decreasing GSK-3*β* and DKK1 proteins and mRNAs levels [37]. Jiang et al. showed a novel anti-inflammatory mechanism induced by EA via *α*7nAChR-mediated inhibition of NLRP3 inflammasome in rats after cerebral ischemic injury [38].

## 3. The Therapeutic Effect of EA for Ischemic Stroke in Clinic

### 3.1. The Effect of EA on Central Nervous System in Stroke Patients

EA at head acupoints in stroke patients may contribute to the stimulation of nerve tissue involved with motion via activating the bilateral cerebral motor areas. Furthermore, in six right-handed stroke patients, EA stimulation at Baihui (GV 20) and right Qubin for twenty minutes may also activate other neural regions, suggesting that injured motor functional reorganization is a neural network behavior, and EA may affect several aspects of neural network so as to further promote motor function recovery [39]. Both exercise and EA may promptly improve somatosensory evoked potential of stroke patients in the recovery stage, and the Bobath therapy in combination with EA stimulation was proved to improve cerebral function in stroke patients [40]. Si et al. suggest that EA may improve the neurological function in patients with acute ischemic stroke [17]. Ho et al. demonstrate that EA exerts beneficial effects in stroke, and it might be a suitable nondrug therapy for mobilization of stem cells in CNS [41]. Ouyang et al. suggest that EA of 2/15 Hz and 100 Hz exerts better benefits in improving brain cell functions and local cerebral blood perfusion than that of EA of 2 Hz according to the results of single photon emission computed tomography (SPECT) [42].

### 3.2. The Effect of EA on Poststroke Psychological Illness following Stroke

Man et al. suggest that the dense cranial EA intervention combined with body acupuncture with 2 Hz at 9 volts for 30 minutes at Baihui (GV 20), Yintang (EX-HN 3), Hegu (LI 4), and Quchi (LI 11) might be a feasible therapy for poststroke neuropsychiatric sequelae [43]. Wu and Liu demonstrate that acupuncture at Taichong (LR 3), Shenting (GV 24), GV20, EX-HN 3, GV26, and LI 4, as an effective and crucial therapy, may effectively improve the symptom of poststroke anxiety neurosis (PSAN). The total effective rate of acupuncture stimulation was 82.35% [44]. Tang et al. suggest that the low-frequency EA treatment at the aupoints of Dazhui (GV 14) and Shenshu (BL 23) exerts similar effect for poststroke insomnia to oral medication of estazolam as a secure and effective therapy [45].

Poststroke depression (PSD) is characterized by anxiety, disordered sleep, hopelessness, and lowered responsiveness and is a common stroke complication [46]. Cai et al. show that EA stimulation might be safe and effective for treating poststroke depression (PSD) in clinic [8]. Acupuncture plus auricular point sticking are effective and safe for poststroke depression (PSD). During course of treatment, acupuncture was applied at Baihui (GV 20), Shenting (GV 24), Sishencong (EX-HN 1), Yintang (GV 29), Neiguan (PC 6), Shenmen (HT 7), Taichong (LR 3), Zusanli (ST 36), Hegu (LI 4), Fenglong (ST 40), and Sanyinjiao (SP 6). This combination treatment may improve the clinical symptoms as evidenced by lowered scores, including the sleep disturbance factor, anxiety/somatization factor, and hopelessness factor [47]. EA plus body acupuncture has positive effect on alleviating PSD and cognitive deterioration after stroke, particularly with electrical stimulation on forehead acupoints [48]. Wrist-ankle acupuncture plus fluoxetine can mitigate the depression symptoms after stroke. Moreover, wrist-ankle acupuncture stimulation can increase the antidepressant effect of fluoxetine [49].

### 3.3. The Effect of EA on Improving Spasticity following Stroke

Spasm is the commonest poststroke complication, and its occurrence rate is 20–40 percent in stroke survivors [50]. Moon et al. demonstrate that EA at Shousanli (LI 10), Waiguan (TE 5), LI 11, and LI 4 may transiently alleviate spasticity following stroke, and repeated EA stimulation may sustain the effect of mediating spasticity [51]. Wu indicates that EA treatment at the nerve trunk may significantly facilitate the limbs functional recovery and reduce the rate of disability at the spastic phase of poststroke hemiplegic patients [52]. In addition, the combined application of EA and acupuncture produced a better effect in improving hand spasm, alleviating hand dysfunction and upregulating the quality of life for patients with stroke compared with simple acupuncture [10]. Furthermore, the combination of EA and rehabilitation therapy plays a critical role in regulating lower limbs spasticity in poststroke patients [53]. Wang et al. show that 6-week EA at Zeqian (EX-UE, A32), Shounizhu (EX-UE), Shaohai (HT3), and Neiguan (PC6) in affected side, combined with standard rehabilitation treatment, may decrease the elbow spasticity of chronic stroke survivors [54]. EA at LI 4, Houxi (SI 3), TE 5, LI 11, LI 10, and Jianyu (LI 15), in combination with muscle strengthening training for 6 weeks, may obviously alleviate spasticity of the wrist joint in chronic stroke patients [55]. Liu et al. show that EA plus strength training may promote motor function recovery and alleviate muscle spasticity for moderate or severe muscle spasticity in chronic stroke patients [56].

### 3.4. The Effect of EA on Improving Limbs Function following Stroke

According to enhancement of the upper limbs function, traditional Chinese acupuncture may be beneficial for improving chronic stroke symptoms in patients [57]. Zhao et al. also show that Jingjin acupuncture at GV 26, GV 20, and PC 6 may effectively enhance daily-life ability via improving subtle activity of hemiplegic hand in the phase of poststroke recovery [58]. However, Yang et al. demonstrated that EA therapy may exert beneficial effect in the upper-extremity function following ischemic stroke and provide a better effect than simple manual acupuncture [59]. Hsieh et al. indicate that EA at GV 20, Fengchi (GB 20), LI 15, LI 11, LI 4, Fengshi (GB 31), Yanglingquan (GB 34), and ST 36 on the affected side, may effectively promote motor function recovery, particularly in upper extremity motor function and in patients with the primary ischemic stroke [60]. EA at LI 15, LI 4, TE 5, and LI 10, combined with exercise training, may improve arms and legs function in poststroke hemiplegia patients [61]. Moreover, Liu and Xiao show that EA at Juci and Tanci may improve nail-bed microcirculation in hemiplegic side of poststroke patients, and the effect of Juci stimulation is better Tanci stimulation [62]. Wang suggests that EA at acupoints of different channels exerts benefits on poststroke hemiplegia patients at different stages of stroke [63].

Chen et al. suggest that EA as a supplemental therapy may exert benefits in apoplexy patients with shoulder subluxation [64]. EA treatment at Jianwaishu (SI 14), Jianzhen (SI 9), Naoshu (SI 10), Binao (LI 14), and Bingfeng (SI 12) with intermittent wave and common rehabilitation therapy exerts a better effect compared to continuous and disperse-dense wave for the treatment of shoulder subluxation, and the combination treatment may effectively prompt shoulder functional recovery and improve subluxation [65]. The combination of EA at LI 15, Jianliao (TE 14), and SI 9 and rehabilitation techniques also may exert benefits in regulating the muscular tension of shoulder joint and the muscles around the scapula and muscle strength and improving the shoulder subluxation [66].

In addition, acupuncture intervention at lateral side of BL 10 associated with scalp points, including Zhenxiapangxian (MS 14) and Dingnieqianxiexian (MS 6), plays a critical role in walking ability and standing balance ability after stroke [67]. EA at bilateral MS 6 plays important role in recovery of nerve defects in the hemiplegic patients following acute ischemic brain injury, enhancing limb motor function and the daily-life activity ability [68]. EA (20 Hz, 2 mA) at GV 20, EX-HN 3, GV 26, LI 4, ST 36, SP 6, and Taichong(LR 3), with cupping at the lumboback, exerts a better effect than medication in relief of fatigue in poststroke patients [69]. Liu et al. indicated that EA at Pishu (BL 20), Shenshu (BL 23), Dachangshu (BL 25), and Qihaishu (BL 24) may elevate the single-foot supporting phase rate in stroke patients [70].

### 3.5. The Effect of EA on Improving Swallowing after Stroke

EA stimulation as a feasible and effective therapy may alleviate swallowing dysfunction following stroke at Chonggu acupoints with deep insertion [71]. EA treatment integrated with swallowing functional training may promote the recovery of swallowing ability in poststroke patients with dysphagia [9]. However, Huang et al. show that either electric stimulation or acupuncture at GB 20, LI 18, three-needles on the forehead, etc. combined with rehabilitation training exerts a better effect compared with simple rehabilitation training. The effect of acupuncture in dysphagia is equal to that of electric stimulation [72]. EA at eight-neck-occiput acupoints exerts a better effect on improving swallowing of medulla oblongata palsy following brainstem infarction compared with the routine acupoints [73]. In addition, Su et al. show that EA at Yamen (GV 15), bilateral GB 20, bilateral Renying (ST 9), bilateral Sanyinjiao (SP 6), bilateral LI 4, and bilateral Fenglong (ST 40), may effectively enhance the spleen, clear phlegm, dredge the channels, clean dampness, bring out resuscitation, increase cerebral blood flow, alleviate brain edema, reduce cerebrovascular spasm, promote anoxic tolerance of neuronal cells, and regulate internal organs functions in patients with poststroke dysphagia [74].

### 3.6. The Effect of EA on Speech Apraxia after Stroke

Speech rehabilitation training associated with the scalp electric acupuncture (2 mA, 50 Hz) in Broca's area under anatomic orientation for four weeks may obviously relieve the speech disorder in with poststroke speech apraxia patients (18 cases with cerebral hemorrhage (lesion of 15 cases in the left basal ganglia, lesion of 1 case in the left frontal temporal and parietal, lesion of 2 cases in the left side of the basal ganglia and thalamus) and 42 cases with cerebral infarction (lesion of 11 cases in left bottom of the base section, 12 cases in left frontotemporal top, 11 cases in left insula temporal lobe, 5 cases in left insula and left ventricle narrator, and 3 cases in left frontal lobe and insular lobe) [75]. Chang et al. demonstrate that the stimulation of Xuanzhong and Tongli acupoints provides a therapeutic effect on the aphasia recovery after stroke via activating several brain regions related to language in poststroke aphasic patients [76]. In comparison with the routine acupoints, EA at eight-neck-occiput points plays a better role on speech disability of medulla oblongata palsy following brainstem infarction [73].

### 3.7. The Effect of EA on Improving Cognition and Memory after Stroke

Chou et al. show that EA at PC6 and Shenmen (HT7) for twenty minutes twice a week for eight weeks may improve the recovery of cognition function and life quality in poststroke patients [77]. Based on the rehabilitation training and conventional medication, EA stimulation at Dingniehouxiexian (MS 7), bilateral Ezhongxian (MS 1), Xuanzhong (GB 39), Dingzhongxian (MS 5), LI 4, Taichong (LR 3), ST 36, Taixi (KI 3), and GB 20 five times per week for eight weeks may promote recovery of memory function and the metabolism of cerebral tissue in the poststroke patients (infarct regions: 19 cases of basal ganglia, 9 cases of lateral ventricle, 1 case of thalamus, and 1 case of brainstem), and it has a better effect compared to medication associated with rehabilitation training [78]. Zeng et al. also show that acupuncture at GV 20, EX-HN 1, GV 24, GV 29, LI 4, LR 3, EX-HN 1, GV 24, and GV 29, five times per week for eight weeks can prompt the recovery of cognitive function and improve daily-life ability in subacute stroke patients with mild cognitive dysfunction on the basis of the traditional therapy and the cognitive rehabilitation training [79].

### 3.8. The Effect of EA on Easing Pain after Stroke

EA exerts effective benefits in well-being and pain control via activating antinociceptive pathway in the brain of patients with a history of ischemia in the left temporoparietal region [80]. EA at LI15 and LI 4, plus either penetration needling or routine acupuncture, may exert benefits in improvement of motion function and alleviation of edema and pain for patients with poststroke shoulder-hand syndrome [81]. Chau et al. show that EA treatment may be effective for patients with poststroke shoulder pain to ease the pain, promote upper limbs function, and improve physical function [82]. EA stimulation at Huatuojiaji points may obviously improve postapoplectic thalamic spontaneous pain [83]. Li et al. indicate that EA at Chize (LU 5), LI 15, TE 14, Quze (PC 3), Jianjing (GB 21), and Shaohai (HT 3), in association with Tuina exerts a better effect on poststroke shoulder pain than comprehensive rehabilitation treatment such as the electrostimulation in patients [84].

### 3.9. The Effect of EA on Improving Urinary Function after Stroke

In comparison with indwelling catheter therapy, EA stimulation at Qugu (CV 2), Zhongji (CV 3), Shuidao (ST 28), Qihai (CV 6), and Guanyuan (CV 4) has a better effect in promoting bladder capacity and attenuating apoplectic urinary incontinence in poststroke patients with urinary incontinence [85]. EA treatment (1 Hz, 15 min) at Sanyinjiao (SP6), Ciliao (BL32), and Pangguangshu (BL28) might be a safe therapy for improvement of urinary function because of the effective effects induced by EA on stroke patients with incomplete bladder emptying [86]. EA at Jianyu (LI 15), Xuehai (SP 10), Shenshu (BL 23), Huiyang (BL 35) may also improve micturition clinical symptom and attenuate urinary incontinence severity in stroke patients [87]. Liu et al. indicate that EA intervention at Huiyang and Baliao provides a beneficial effect in alleviating detrusor overactivity after stroke by markedly mitigating symptoms of lower urinary tract, improving bladder compliance and cystometric capacity, reducing upper urinary tract injury risk, and alleviating pressure of detrusor leak point [88].

### 3.10. The Effect of EA on Improving Constipation after Stroke

Abdominal EA treatment at Daheng (SP 15), Fujie (SP 14), Tianshu (ST 25), Shuidao (ST 28), etc. may effectively improve poststroke constipation and accelerate gastrointestinal movement in patients with stroke [89]. Wang et al. show that basic comprehensive treatment in combination with EA at the point of Zusanli (ST 36) and Tian-shui (ST 25) plays a key role in prevention and treatment of constipation symptom in the acute phase of ischemic stroke [90].

### 3.11. The Effect of EA on Other Aspects after Stroke

Fu et al. show that EA at Jianyu (LI 15), Biguan (ST 31), Hegu (LI 4), Taichong (LR 3), Quchi (LI 11), Yanglingquan (GB 34), and Shenshu (BL 23), combined with dissolve-stasis herbs, rehabilitation training, and active-blood herbs, may be effective for treating ischemic stroke in clinic [91]. Electrospoon needles or electrofiliform needle may effectively promote motor dysfunction and daily-life ability in ischemic stroke patients [92]. Qian et al. show that acupuncture intervention at Jiquan (HT 1), Quchi (LI 11), Hegu (LI 4), Huantiao (GB 30), etc., twice per day in convalescence of cerebral infarction may exert more benefits than once per day in patients [93]. Li shows that EA at acupoints of either Yin Meridians or Yang Meridians may induce protection in poststroke patients [94]. Wong et al. suggest that EA via adhesive surface electrodes combined with appropriate rehabilitation therapy is an effective and convenient treatment for stroke patients [95]. EA and acupoint injection may significantly elevate daily-life ability and improve the neural function for the ischemic stroke patients, exerting a better effect than that EA alone [96]. Li et al. indicate that the combination therapy of EA at GV 20, Shenzhu (GV 12), Tianding (LI 17), LI 10, Biguan (ST 31), and Fenglong (ST40) and intracarotid drug injection may increase the cerebral blood vessels elasticity, promote vasodilation, and elevate the cerebral blood flow, contributing to sufficient supply of blood and oxygen and recovery of ischemic brain function following cerebral infarction [97].

Pei et al show that EA stimulation plays an important role in improving life quality and in health care, social services, and daily living ability of patients in acute stage EA stimulation at LI 4, LI 10, LI11, LI15, SP 6, Fenglong (ST 40), ST 36, and DU20 may prompt motor function recovery and then improve the living activities in the early stage of stroke [98]. Wang et al. suggest that EA treatment is important in improving the nervous dysfunction deficits following four-week intervention and enhancing the daily-life activity level following six-month follow-up visit, and systematic acupuncture treatment may alleviate the occurrence rate of secondary apoplexy in patients [99].

## 4. Conclusion

**Figure 1:**
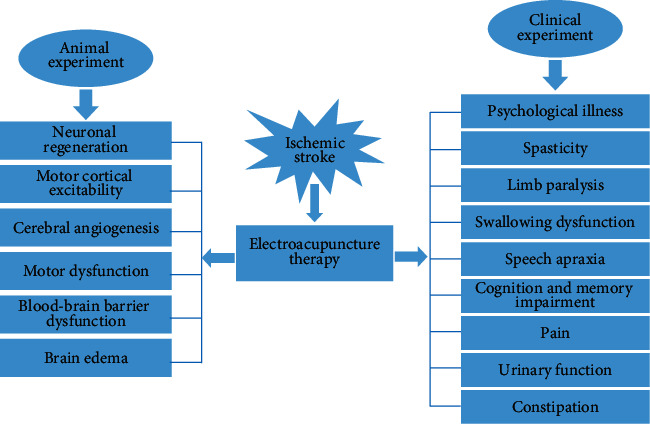
Electroacupuncture could promote neuronal regeneration, increase motor cortical excitability, improve cerebral angiogenesis, reduce motor dysfunction, decrease brain edema, and alleviate the impairment of blood-brain barrier according to the results of animal experiments. Meanwhile, electroacupuncture could improve a series of dysfunctions following stroke, including constipation, urinary function, pain, cognition and memory impairment, speech apraxia, swallowing disability, limb paralysis, spasticity, and psychological illness according to the results of clinical trial.

In summary, as demonstrated in Figure 1, EA treatment or preconditioning may play an important role in alleviating edema, easing pain, enhancing cerebral blood flow and daily-life ability, improving cognition and memory function, speech function, swallowing function, motor function, as well as nerve, intestinal, and urinary system. In addition, EA stimulation combined with other common rehabilitation treatment might exert better effect for treatment of stroke than EA alone. EA with high frequency or long duration may elicit effective improvement in apoplectic patients. The effect of EA stimulation also involves acupoints, intensity, and interval of stimulation. All of those mentioned above provide a potential treatment strategy for treating apoplectic patients in clinic.

## Data Availability

This is a review article with no underlying data.
